# Component analysis of somatosensory evoked potentials for identifying spinal cord injury location

**DOI:** 10.1038/s41598-017-02555-w

**Published:** 2017-05-24

**Authors:** Yazhou Wang, Guangsheng Li, Keith D. K. Luk, Yong Hu

**Affiliations:** 1 Spinal division, Department of Orthopaedics, Affiliated Hospital of Guangdong Medical College, Guangdong, 524001 China; 2 Department of Orthopaedics and Traumatology, The University of Hong Kong, Pokfulam, Hong Kong; 3 Shenzhen Key Laboratory for Innovative Technology in Orthopaedic Trauma, Department of Orthopaedics and Traumatology, The University of Hong Kong-Shenzhen Hospital, Shenzhen, 518060 China

## Abstract

This study aims to determine whether the time-frequency components (TFCs) of somatosensory evoked potentials (SEPs) can be used to identify the specific location of a compressive spinal cord injury using a classification technique. Waveforms of SEPs after compressive injuries at various locations (C4, C5 and C6) in rat spinal cords were decomposed into a series of TFCs using a high-resolution time-frequency analysis method. A classification method based on support vector machine (SVM) was applied to the distributions of these TFCs among different pathological locations. The difference among injury locations manifests itself in different categories of SEP TFCs. High-energy TFCs of normal-state SEPs have significantly higher power and frequency than those of injury-state SEPs. The location of C5 is characterized by a unique distribution pattern of middle-energy TFCs. The difference between C4 and C6 is evidenced by the distribution pattern of low-energy TFCs. The proposed classification method based on SEP TFCs offers a discrimination accuracy of 80.2%. In this study, meaningful information contained in various SEP components was investigated and used to propose a new application of SEPs for identification of the location of pathological changes in the cervical spinal cord.

## Introduction

Somatosensory evoked potentials (SEPs) can be generated in response to electrical stimulation of peripheral nerves such as the median nerve or posterior tibial nerve. The SEP signal recorded from the scalp consists of a series of electrophysiological waves generated and mediated at different sites along the somatosensory pathway^1–3^. In clinical applications, SEPs have been used only as an additional diagnostic tool for evaluating the functional integrity of the nervous system^4–6^. For example, intraoperative cortical SEP monitoring uses the amplitude and latency of the main component as a measure of the possible neurological deficits that result from the surgery^7, 8^. An abnormal SEP result can only demonstrate that a dysfunction exists within the somatosensory pathway without offering any information on the pathological changes following insults to the sensory nerve fibers.

Previous studies involving human patients and monkey subjects^9–14^ showed that a series of smaller components separate from the main component in the SEP signal are likely to have various origins along the somatosensory pathway. This finding infers that these small components might correspond to distinct anatomical structures within the pathway. Based on this finding, we propose that SEPs not only produce information related to the functional integrity of the ascending sensory tracts but also have the potential to reflect the anatomic site of damage to the pathway that leads to pathological changes of the nerve fibers.

A previous work^15^ reported that changes in the distribution patterns of SEP time-frequency components (TFCs) might be related to the locational difference of neurological deficits following spinal cord injury (SCI). A follow-up study^16^ further found that these location-related patterns are stable in both acute and chronic SCI. The results of these two previous studies implicated (but did not verify) the usefulness of SEPs in identifying the location of neurological deficits in the cervical spinal cord, which motivated us to raise the hypothesis that SEP TFCs could be used as an indicator of the location of SCI, resulting in pathological changes in the spinal cord. This method is novel and significant if we can prove the usefulness of SEP TFC in identification of the location of cord pathological changes. The current study was conducted to test this hypothesis by developing a classification method for identifying the location of pathological changes after SCI using distribution patterns of SEP TFCs. This study is the first in the field of clinical electrophysiology to explore the new use of SEPs in identifying the pathological site in a cervical spinal cord.

This study uses the distributions of SEP TFCs of various pathological sites of SCI to develop a classification system that uses the machine learning technique. This technique learns from the stable patterns of SEP TFCs (training data) to build a classifier that can make predictions on an input SEP signal with respect to the pathological status of the cervical spinal cord (intact or not) and the location of pathological changes.

## Methods

### Materials

All animal experimental protocols were approved by the committee on the use of live animals for research at Guangdong Medical College. All experimental procedures were conducted in strict compliance with the institutional guidelines for the care and use of laboratory animals in the Affiliated Hospital of Guangdong Medical College at Guangdong Province, China. In this study, 36 adult Sprague-Dawley (SD) rats (250–300 g) underwent implantation of compressors at various locations in the spinal cord (12 rats at C4, 12 rats at C5, and 12 rats at C6). Before and 2 weeks after the surgical procedures, SEPs were recorded from all rats. Another 12 rats underwent sham compression, which means that surgical procedures except for compressor implantation were performed, and SEP recordings were also collected from these rats 2 weeks after surgery.

### Experimental procedures

Animals were operated on under inhalant anesthesia with isoflurane (3% for induction and 1.5% for maintenance). After the occipital and nuchal areas were shaved and prepared with iodine solution, a skin incision was made to expose the laminae C3–C7 on one side with removal of ligamentum flavum between them through a posterior approach under microscopy. A small space surrounding the facet was opened with laminotomy and enlarged with natural flexion of the spine. The dura underneath was carefully separated from the laminae to prevent leakage of cerebrospinal fluid. A water-absorbing polymer was carefully inserted into the spinal canal at the planned compression location (C4, C5, or C6). The polymer^17, 18^ (size 1 × 2 × 1 mm) consisted of 3% agarose gel (Amresco LLC, Solon, OH, USA) that was dried for 8 hours by vacuum dryer and could absorb liquid in the spinal canal to reach its maximum expansion (four-fold in volume) in 2 hours. Once saturated with liquid, the volume remains stable at the maximal volume for 6 months^18^. This material could therefore produce a chronic course of compression on the cervical spinal cord in the rat. No implantation was performed for the sham compression. SEP monitoring was performed to ensure no acute spinal cord injury occurred during operation. After implantation, the incision was closed in layers, and the animals were given an intramuscular injection of penicillin to prevent infection.

### SEP data collection

For the rats with spinal cord compression, two SEP recordings were collected from a single animal. One recording was collected before the surgical procedures, and the other recording was collected two weeks after compressor implantation at various locations (C4, C5 or C6). Therefore, data for classification included 36 SEP recordings in the normal condition, referred to as the normal group, and 36 SEP recordings in the injured condition, referred to as the C4 group (12 recordings), C5 group (12 recordings), and C6 group (12 recordings). At the time point of two weeks, pathological changes caused by SCI had become relatively stable and had not begun to spread beyond the target level into the other adjacent levels. Only one SEP recording was collected from each of the 12 rats with sham compression (also two weeks following surgery). These 12 recordings were known as the sham group. SEP responses were elicited by a constant current stimulator at the rate of 4.1 Hz applied to the rat forelimb. The stimulus intensity was set to cause a mild twitch of the forelimb. Consistent, large, and well-defined cortical SEP waves were obtained from a needle electrode inserted subcutaneously above the rat somatosensory cortex. During SEP recording, rodent subjects were held under inhalant anesthesia with isoflurane (3% for induction and 1.5% for maintenance). The recorded signals were amplified 2,000 times with a bandpass filter of 10–2000 Hz (Zhuhai Yiruikeji Co., Ltd., China). Each SEP recording is the result of an average of 200 responses. It should be noted that only one SEP recording was taken from a single animal.

### Pathological evaluations

After experiments, the rats were sacrificed by overdose of intravenous sodium pentobarbital and perfused with 50 ml heparin-saline through the ascending aorta and 300 ml formalin-picric solution (4% formaldehyde, 0.4% picric acid in 0.16 mol/L phosphate buffer, pH = 7.4). The entire cervical spinal cord was carefully harvested and fixed with 4% phosphate buffer liquid in formaldehyde solution for another 72 h. The cords were embedded in paraffin. Transverse sections (8 μm) were prepared for pathological examination by hematoxylin-eosin (H&E) staining and myelin sheath evaluation by solochrome cyanine (SC) staining in the white matter of the spinal cord. The slides were analyzed under a microscopic imaging system (FV-1000, Olympus, Japan). The intensity in blue color (Image-Pro Plus 6.0, Media Cybernetics, Inc.) indicated the content of myelin, and neurons were identified by locating the nuclei and abundant Nissl bodies within the perikarya in the gray matter.

### Identification and classification of SEP time-frequency components

**Figure 1:**
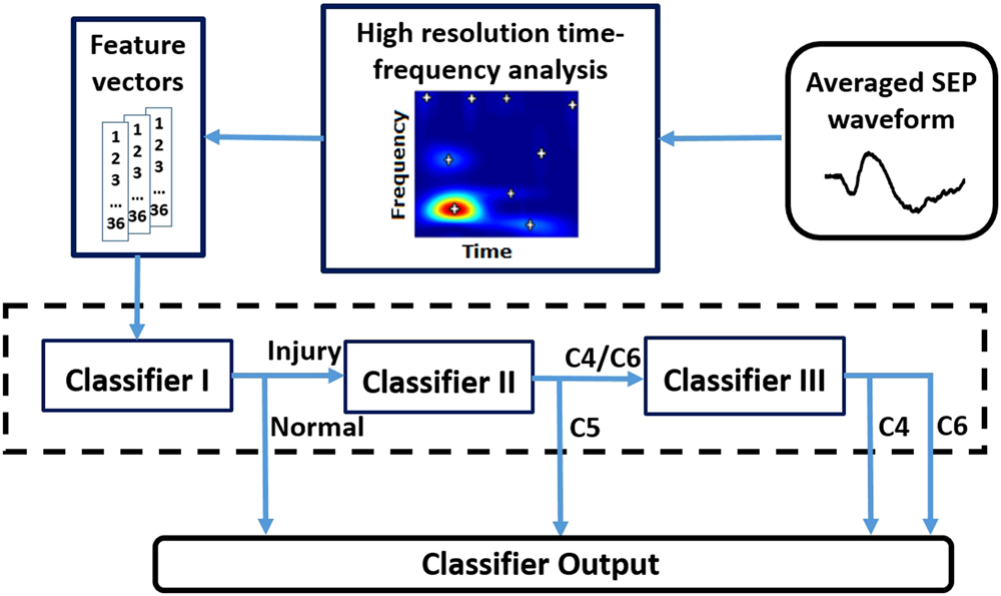
Architecture of the SEP-based classification system for identifying the location of pathological changes caused by SCI.

In this study, a high-resolution time-frequency decomposition algorithm, i.e., matching pursuit (MP), was used to decompose the averaged SEP signals into a number of time-frequency components (TFCs) (white crossings in Fig. 1) with certain parameter descriptions. This algorithm was applied to analysis of rat SEPs in previous studies^16, 19^. The parameters of these TFCs included time, frequency and power and were combined into feature vectors. These vectors were subsequently used as input data that were fed into the proposed classification method together with labels corresponding to various conditions. Joint probability density estimation was also conducted to obtain the stable distributions of these TFCs. At each point in the time-frequency domain, the probability density function (PDF) was calculated to reflect the distribution density of the TFCs.

All of the decomposed TFCs were divided into three categories according to their relative energy: 1) the TFC with the highest energy in each SEP signal was defined as a ‘high-energy TFC’; 2) other TFCs, other than the high-energy TFC, with relative energies greater than 2% were defined as ‘middle-energy TFCs’; and 3) the remaining TFCs were defined as ‘low-energy TFCs’. This categorization (with the relative energy threshold of 2%) was based on a previous study^19^, and we found in the current study that TFCs of the three injury groups showed distinct distribution patterns above (C5 vs. C4&C6) and below (C4 vs. C6) this threshold that could be used as useful prior knowledge for the following classification process.

Based on the distributions of these TFCs in the time-frequency domain, a supervised learning technique, i.e., support vector machine (SVM), was adopted to infer the spinal cord status for a given condition (normal or damaged at C4/C5/C6). SVM has been used in the field of electrophysiology for many years^20, 21^. A total of 72 SEP recordings were used in SVM classification, namely, 36 recordings in the normal group and 36 recordings in three injury groups (each injury group contained 12 recordings). The datasets of the four groups were separately recorded without any crosstalk and therefore could be considered independent of each other among the groups.

In this study, the classification process was conducted in three steps that respectively correspond to the three sub-classifiers (Fig. 1). Classifier I was used to distinguish normal SEPs from the injured. Classifier II was responsible for differentiating the C5 group from the other injury groups. Classifier III was used to discriminate between the C4 group and C6 group.

Because inappropriate selection of the parameters of the SVM algorithm might lead to the over-fitting/under-fitting problem^20^, cross-validation was performed to investigate the optimal parameters and better gauge the average performance of the classifiers. Detailed information on the cross-validation and parameter optimization process is introduced in the following paragraphs.

Use of all data to train a classifier and evaluation the performance of the classifier with the same data can cause the over-fitting problem. In this study, a 10 × 10-fold cross-validation was used to overcome this problem and assess the prediction accuracy of the proposed classifier, which means that the cross-validation was iterated with ten random dataset splits. During the process of 10 × 10-fold cross-validation, the entire dataset (all 72 recordings) was randomly partitioned into 10 subsets. Among all subsets, eight subsets contained 7 recordings and the other two subsets contained 8 animals because the entire dataset (72 recordings) could not be equally partitioned into ten subsets. Of the ten subsets, a single subset was retained as a test set, and the remaining nine subsets were grouped together to form a training set to construct a predictor. The cross-validation process was repeated ten times, with each of the ten subsets used as the validation data exactly once. The ten results from the folds were averaged to produce a single estimation. The entire process was repeated for ten different runs, and an average of the ten estimations was calculated as the final cross-validation accuracy.

In this research, the radial basis function (RBF) kernel was applied as the kernel function of SVM. Two important parameters were applied, i.e., C (regularization parameter) and gamma (kernel parameter), which needed to be properly adjusted to obtain the optimal performance. To search for the optimal values of C and gamma, a grid search and 10 × 10-fold cross-validation were used on the training data. Principally, all pairs of (C, gamma) were tested, and the parameter pair with the highest mean accuracy over all cross-validation iterations was selected. The grid search ranges were log_2_(C) = −2:20 and log_2_(gamma) = −14:10 with an interval of 1.

## Results

### Pathology

The location of pathological changes caused by SCI was verified using histological and histochemical evaluations.

**Figure 2:**
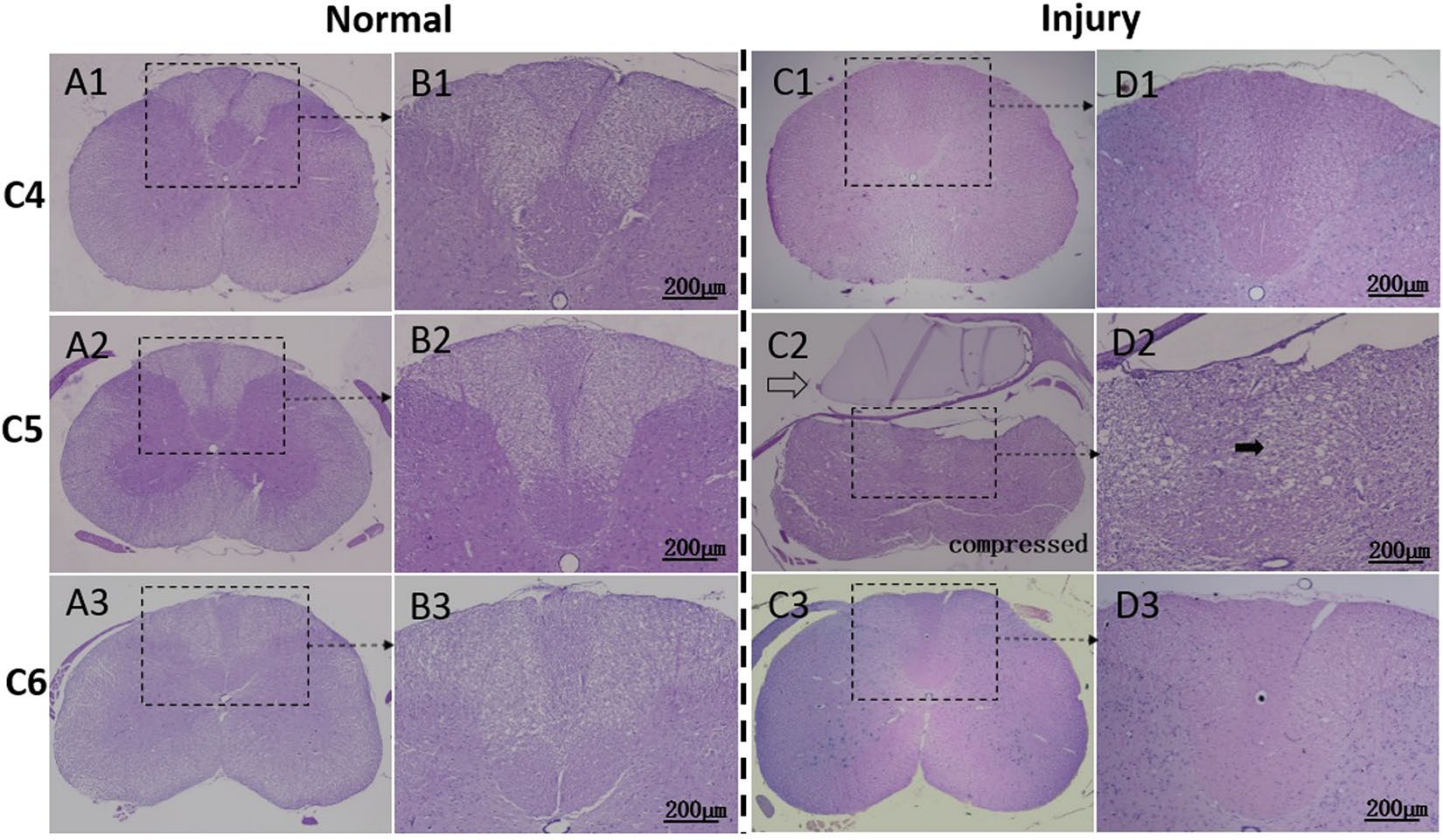
Histological features of normal and injured cord by hematoxylin erosion (H&E) staining. The normal cord with sham compression showed structural integrity at C5 level (A_2_) as well as the adjacent C4 (A_1_) and C6 (A_3_) level. Butterfly-like gray matter could be clearly identified (A_1–3_). Distinguished neural fiber tracts derived from dorsal horn (B_1–3_) could be observed; The compressor (hollow arrow) at C5 level expanded its volume, induced compression injury to the cord, and led to notable structural deformation and disorganization (C_2_ and D_2_). Evident tissue loss and diffuse vacuolization (solid arrow) of the posterior funiculus in white matter were verified at the C5 injury level (D_2_). By comparison, the adjacent levels, C4 (C_1_, D_1_) and C6 (C_3_, D_3_), with integral and organized structures, which were similar with the normal cord (A_1–3_ and B_1–3_), showed no distinguishable pathological changes.

Histological evaluation results: In the intact spinal cord, the tissue of the posterior funiculus was clearly distinguishable, and the sensory neural fiber tracts derived from sensory neurons of the dorsal horn in the gray matter to the white matter could be clearly identified. After two weeks of compression, the tissue loss of the cord, especially in the posterior funiculus, could be clearly viewed as vacuolization (solid arrow in Fig. 2D_2_). Edema and sparse microstructure as well as disorganized neural fiber tracts could be observed in the posterior funiculus. Posterior funiculus fibers of white matter appeared to be broken or vanished (Fig. 2D_2_). The number of large motor neurons in the ventral horn decreased significantly in the injury condition (Table 1). In the neuron, loss of cytoplasm, karyopyknosis of the nucleus, and decrease of neuronal synapse could be observed.

**Table 1:** Large motor neuron counting and myelin staining intensity comparison between normal and injured cords.

	Normal	Injured
The number of large motor neuron	20.8 ± 2.2	4.6 ± 1.0*
Myelin staining intensity^1^	87 ± 4	35 ± 2*

^*^Compared with normal condition, p < 0.05 (Student’s t-test);

^1^measured by Image-Pro Plus 6.0 (Media Cybernetics, Inc.).

**Figure 3:**
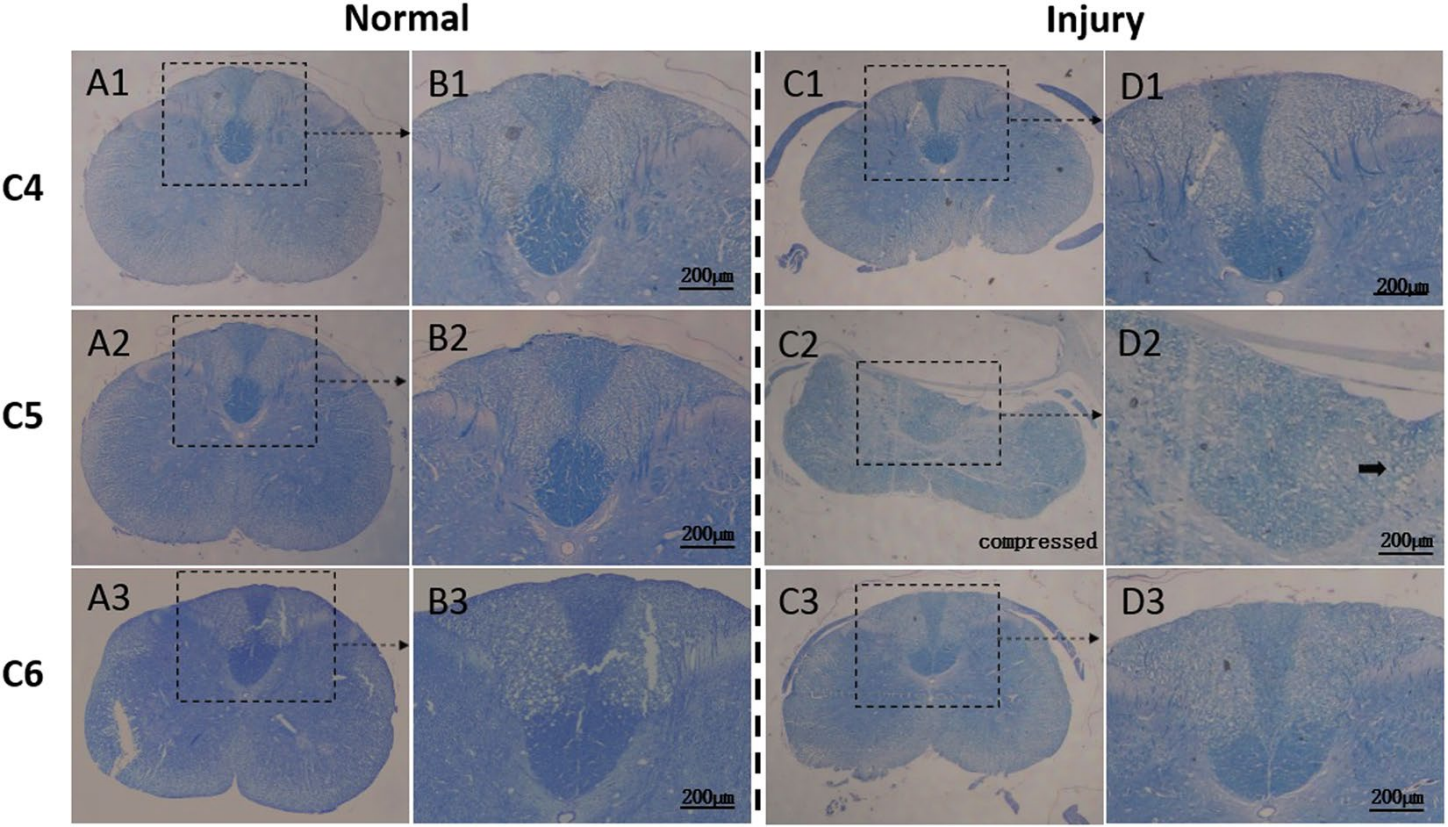
Histochemical features of normal and injured cord by solochrome cyanine staining. The normal cord with sham compression showed integral overview at C5 level (A_2_) as well as the adjacent C4 (A_1_) and C6 (A_3_) level. The myelin sheath of axon was stained with blue and had dense structure. The blue neural fiber tracts derived from the dorsal horn of the cord could be clearly identified (B_1–3_); Two weeks after cord compression, evident structural deformation and tissue loss of the posterior funiculus were demonstrated at the compressive epicenter (C_2_). The blue dyeing neural fibers were not discernible, axon network was broken, and the blue staining in myelin of neural fiber was decolored (D_2_). Moreover, vacuolar degeneration (solid arrow), which implicated axon demyelination, was confirmed (D_2_). By contrast, the adjacent levels, C4 (C_1_, D_1_) and C6 (C_3_, D_3_), showed intact morphology, organized neural fiber tracts and networks, which were similar with the normal cord (A_1–3_ and B_1–3_).

Histochemical evaluation results: In the intact spinal cord, the myelin sheath and the nerve fiber tracts were shown in dark blue and had dense structure. The sensory nerve fiber tracts derived from sensory neurons of the dorsal horn in the gray matter to the white matter could be clearly distinguished. Two weeks after implantation of compressor, evidence of structural deformation and tissue loss of the posterior funiculus appeared. Neural fibers were also disorganized, the axon network was broken, and the blue staining in the myelin of neural fibers decreased (Fig. 3D_2_). Vacuolar degeneration was observed around the axons (solid arrow in Fig. 3D_2_), and a significant decrease was noted in the thickness and blue staining intensity (Table 1) of the myelin sheath, suggesting a demyelination change of the neural fiber (Fig. 3D_2_).

It is worth noting that the compression resulted in no change at the levels that were adjacent to or remote from the compressed segments. In Figs 2 and 3, the exact pathological site was verified by histological and histochemical evaluations. In C_2_ and D_2_, pathological changes at the compressed cord level C5 were shown. The injured cord showed obvious deformation and structural disorder (e.g., vacuolization indicated by solid arrows) at C5, whereas the adjacent levels (C4: C_1_ and D_1_; C6: C_3_ and D_3_) showed similar integral and organized structures with the normal cord. Other levels that were remote from the compressed level, such as C3 and C7, also remained intact after compression, as confirmed by histological evaluation. The conclusion could therefore be drawn that compression-related changes were restricted to the cord levels where compression was applied.

### SEP waveforms and time-frequency analysis

**Figure 4:**
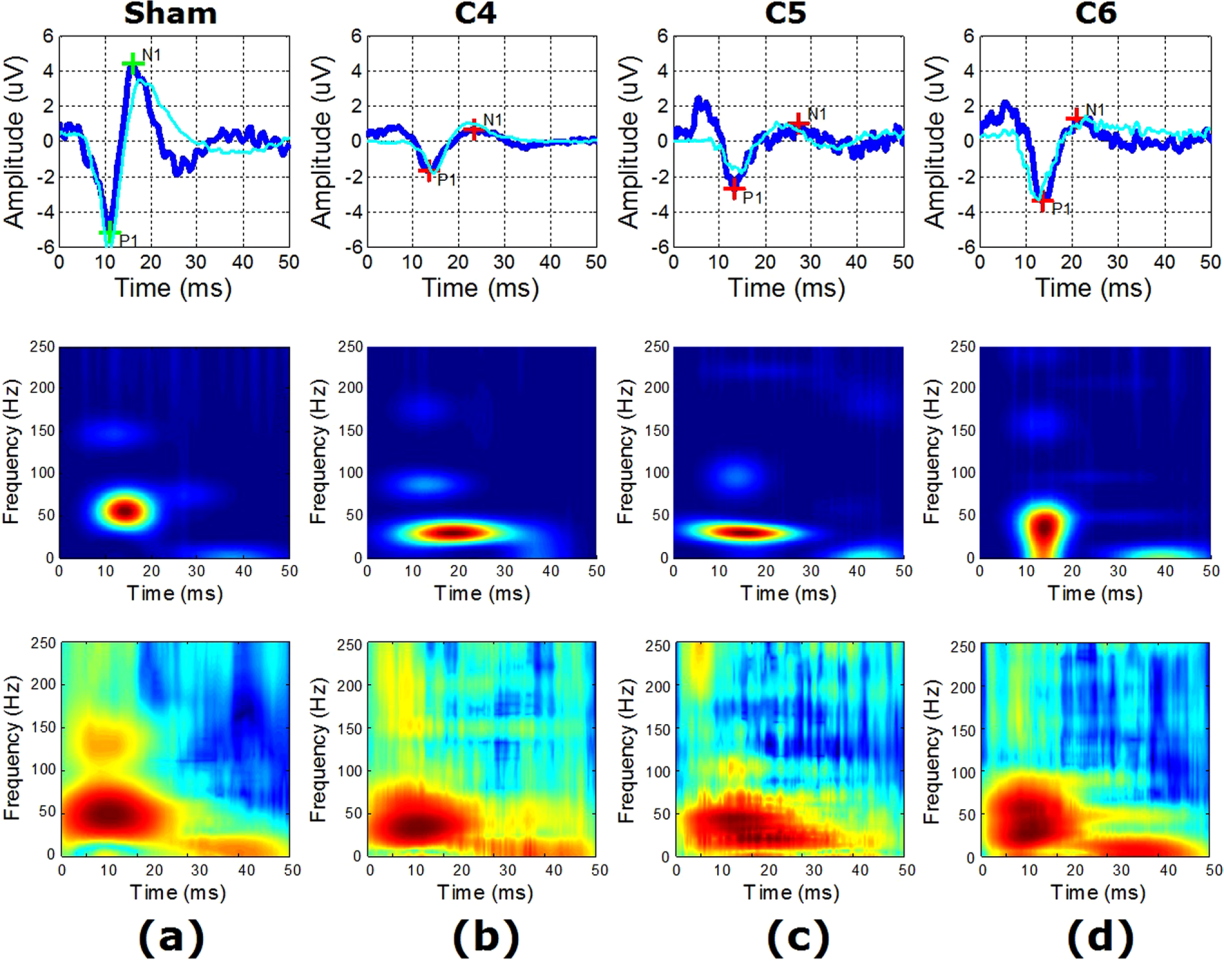
Example (from four representative animals) and averaged (over all animals for each condition) SEP waveforms and time-frequency distributions. Columns (conditions): (**a**) normal, (**b**) injury at C4, (**c**) injury at C5, (**d**) injury at C6. First row: SEP waveform (blue) from an example animal for each condition with P1-N1 peaks marked as green (normal) and red (injury) crosses, and SEP waveform (cyan) averaged over all animals for each condition. Second row: MP-based time-frequency distribution corresponding to the example waveform in the first column. Third row: MP-based time-frequency distribution averaged over all animals for each condition corresponding to the averaged waveform in the first column.

After 2 weeks of compression, a decrease in amplitude and an increase in latency of SEP could be clearly observed (first row in Fig. 4). One dominant peak (high-energy TFC) was noted (second and third row in Fig. 4) accompanied by a series of smaller components (middle- and low-energy TFCs). The SCI led to a reduction in frequency of the high-energy TFC and visibly altered the distribution patterns of the other TFCs. More importantly, obvious differences among different injury levels could be visualized in the SEP time-frequency distributions.

### Classification

The high-energy TFC (main component) of the SEP signal has been used to assess the integrity of the spinal cord^22, 23^. This study compared the characteristics of the high-energy TFCs in normal group and sham group. The time, frequency, and power (mean ± standard deviation) values of the normal group high-energy TFCs were 12.9 ± 4.2 ms, 50.7 ± 8.2 Hz, and 50.4 ± 28.5 μV^2^, respectively. Comparatively, in the sham group, the high-energy TFCs were 13.6 ± 4.3 ms in time (p = 0.62, Student’s t-test), 49.7 ± 7.6 Hz in frequency (p = 0.66, Student’s t-test), and 49.1 ± 25.3 μV^2^ in power (p = 0.88, Student’s t-test). These observations indicate that after surgical procedures without compressor implantation, the spinal cords of the rats remained intact and showed no significant difference from the normal condition in the time-frequency features of SEPs. Therefore, the following classification processes did not involve the sham group recordings.

**Figure 5:**
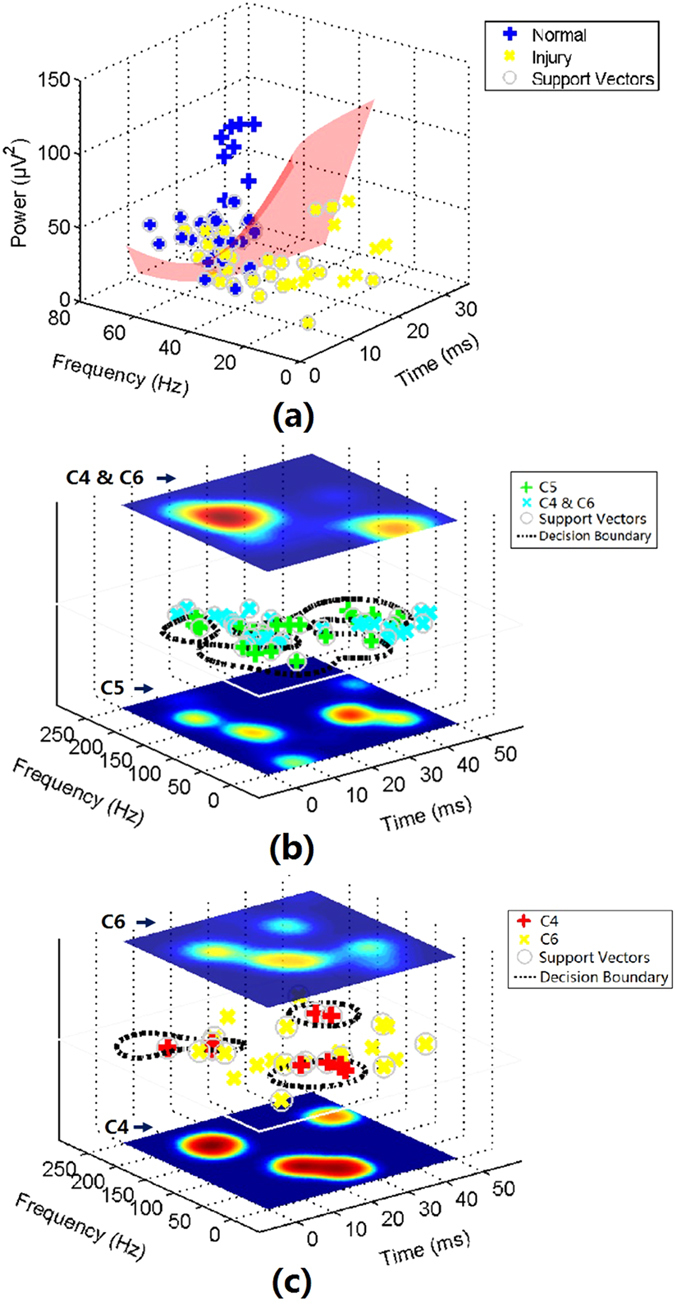
Distributions of high-energy, middle-energy, and low-energy TFCs and illustrations of SVM-based classification. (**a**) Distribution of high-energy TFCs in the time-frequency-power space. Red surface indicates the decision boundary of Classifier I for differentiating between normal group TFCs and injury group TFCs; (**b**) Distribution of middle-energy TFCs of the three injury groups in the time-frequency domain (center plane). Dashed curves indicate the decision boundaries of Classifier II for differentiating between C5 group TFCs and C4&C6 group TFCs. PDFs of TFCs from these two classes are shown in the bottom (C5) and top (C4&C6) plane; (**c**) Distribution of low-energy TFCs of the three injury groups in the time-frequency domain (center plane). Dashed curves indicate the decision boundaries of Classifier II for differentiating between C4 group TFCs and C6 group TFCs. PDFs of TFCs from these two classes are shown in the bottom (C4) and top (C6) plane.

The high-energy TFCs of normal SEPs showed distinct distribution regions in the time-frequency-power space compared with those in the injury groups (Fig. 5(a)). After chronic SCI, a significant decrease was noted in frequency (34.7 ± 12.3 Hz, p < 0.01, Student’s t-test) and power (28.5 ± 14.2 μV^2^, p < 0.01, Student’s t-test) of the high-energy TFCs, whereas the time was only slightly prolonged (15.1 ± 6.7 ms) with no significant difference from the normal state. Based on this finding, Classifier I was trained to distinguish the high-energy TFCs between the normal and injured conditions.

**Figure 6:**
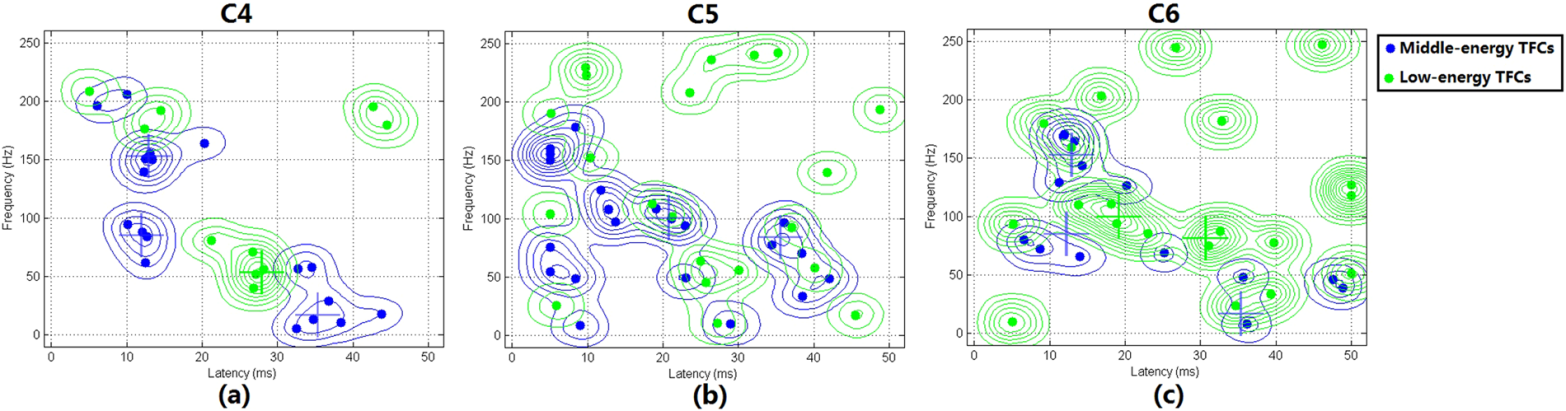
Distribution patterns of the middle- and low- energy TFCs of the injury groups in the time-frequency domain. Local peaks of the PDF distribution of middle- and low-energy TFCs are marked with crosses.

Using the middle-energy TFCs, a ‘one-stop’ classifier could not be constructed to distinguish the three injury groups (C4, C5, and C6) because a considerable portion of the middle-energy TFCs in C4 and C6 group showed similar distributions (Fig. 6(a),(c)). The middle-energy TFCs in the C5 group were located separately from the others (Fig. 6(b)). Based on the distinctive distribution pattern of the C5 group TFCs, Classifier II was trained to differentiate between the middle-energy TFCs in the C5 group and those in the other two injury groups. The dashed curves in Fig. 5(b) represent the decision boundary of SVM algorithm.

The difference between C4 and C6 group was primarily reflected in the distribution pattern of low-energy TFCs (Fig. 6(a),(c)). It was found that low-energy TFCs in both the C4 and C6 groups had scattered distributions in the time-frequency domain. However, their distribution regions were separated from each other and thus could be classified using Classifier III (Fig. 5(c)).

A 10 × 10-fold cross-validation was used to determine the optimal values of SVM parameters, C and gamma, and evaluate the performance of the complete classification method. The optimal pairs of log_2_(C) and log_2_(gamma) of the three sub-classifiers are: (1, −12) for Classifier I; (2, 6) for Classifier II; (8, 2) for Classifier III. With the optimal parameters, Classifiers I, II and III were found to achieve averaged cross-validation accuracies of 78.9%, 80.7% and 86.0%, respectively.

The averaged accuracy of the 10 × 10-fold cross-validation was 80.4 ± 2.0%, ranging from 76.8% to 83.6% (in the ten iterations). In other words, with an input of SEP signal in the four groups (normal, C4, C5, and C6), the system could classify it into one of the groups with an accuracy of 80.4%.

### Important TFC distribution regions

As shown in Figs 5 and 6, the TFCs of the three injury groups appeared in a number of separated regions in the time-frequency domain with certain occurrence rates. This phenomenon was also observed in a previous study that analyzed SEP components recorded from rats^19^. The variability of TFCs in each region was represented by the standard deviation, and the importance of these regions was measured using the value of the PDF and the rat occurrence rate.

In the PDF distribution, all local peaks were detected. For the middle-energy TFCs of the C4 and C6 groups, by checking the PDF values of all local peaks, it was found that only three peaks had PDF values greater than 80% of the maximum PDF value (blue crosses in Fig. 6(a) and (c)), whereas values of the other local peaks were less than 20%. Near each of these peaks, TFCs concentrated within a region where they had similar temporal and frequency features. For each region, the coverage in the time-frequency domain, the location of the corresponding local PDF peaks, the mean and standard deviation of the TFCs inside, and the rat occurrence rate can be found in Table 2.

**Table 2:** Important distribution regions of middle-energy TFCs of C4 and C6 group.

	T-F ranges	Local PDF peak	TFCs (Mean ± SD)	Rat occurrence rate
Region 1	0–25 ms,	13.1 ms,	13.1 ± 3.8 ms,	58.3% (14/24)
	125–225 Hz	153.0 Hz	158.7 ± 23.4 Hz	
Region 2	0–25 ms,	11.7 ms,	10.9 ± 2.6 ms,	33% (8/24)
	50–125 Hz	79.5 Hz	78.1 ± 12.0 Hz	
Region 3	25–50 ms,	36.2 ms,	37.2 ± 6.7 ms,	50% (12/24)
	0–75 Hz	13.0 Hz	33.5 ± 22.3 Hz	

Similarly, for the middle-energy TFCs of the C5 group, two main peaks were found in the PDF distribution (blue crosses in Fig. 6(b)). Near each of the two peaks, a region occurred in which SEP TFCs could be found with high distribution density. The detailed characteristics of these regions can be found in Table 3.

**Table 3:** Important distribution regions of middle-energy TFCs of C5 group.

	T-F ranges	Local PDF peak	TFCs (Mean ± SD)	Rat occurrence rate
Region 1	10–25 ms,	21.5 ms,	16.9 ± 4.7 ms,	58.3% (7/12)
	75–125 Hz	100.5 Hz	105.3 ± 10.8 Hz	
Region 2	30–45 ms,	35.5 ms,	37.9 ± 2.9 ms,	41.7% (5/12)
	25–100 Hz	83.0 Hz	65.6 ± 24.5 Hz	

The locational differences (in the time-frequency domain) of the regions in Tables 2 and 3 between the C5 group and C4 and C6 groups was particularly important in the performance of Classifier II.

For the low-energy TFCs of the C4 group, only one main peak was noted in the PDF distribution (green cross in Fig. 6(a)), around which SEP TFCs were densely distributed within a region. Coverage of the region ranged from 20 to 30 ms in time and from 0 to 100 Hz in frequency. The position of the peak occurred at 27.8 ms and 55.0 Hz. The time and frequency (mean ± standard deviation) of the TFCs were 25.7 ± 2.6 ms and 66.4 ± 20.8 Hz, respectively.

For the low-energy TFCs of C6 group, two main peaks were found in the PDF distribution (green crosses in Fig. 6(c)). Near each of the two peaks, TFCs were located within a region where they showed similar temporal and frequency features. The detailed characteristics of these regions can be found in Table 4.

**Table 4:** Important distribution regions of low-energy TFCs of C6 group.

	T-F ranges	Local PDF peak	TFCs (Mean ± SD)	Rat occurrence rate
Region 1	5–25 ms,	18.7 ms,	18.5 ± 3.8 ms,	50.0% (6/12)
	75–125 Hz	100.5 Hz	100.0 ± 12.2 Hz	
Region 2	30–50 ms,	30.6 ms,	41.3 ± 7.6 ms,	50.0% (6/12)
	0–100 Hz	83.0 Hz	53.9 ± 21.9 Hz	

The locational difference (in the time-frequency domain) of the above three regions between the C4 group and C6 group was particularly important in the performance of the Classifier III.

All of these distribution regions of the SEP TFCs in various injuries groups contributed greatly to the established classifiers, and the TFCs within these groups were closely related to the pathological sites of SCI. The TFCs outside of these regions were more likely to be unassociated with the location of pathological changes.

## Discussion

The conventional SEP analysis technique^6, 15^ uses only time domain parameters such as latency and amplitude and the main component (corresponding to the high-energy TFC in this study) of the SEP waveform. A series of small time-frequency components, neglected in the conventional understanding of SEP morphology, have been found to contain information on the location of neurological deficits caused by SCI^16, 24^, which suggested that SEP TFCs might be used as an indicator of the exact location of pathological changes following SCI. Based on this information, the current study further explored the application of SEP in locating lesions within the somatosensory pathway using the time-frequency distribution patterns of these small components.

In this study, all components of SEP in the time-frequency domain were compared in three energy scales. High-energy TFCs corresponded to the main component (main peak) in previous studies^22, 23^, which had been found to be useful in reflecting whether an injury is present in the spinal cord^16^. Middle-energy and low-energy TFCs, i.e., the small components mentioned above, were found to be useful in locating the site of injury in the spinal cord in the current study. Using these two types of TFCs, SEPs could be applied to distinguish among the locations of C4, C5 and C6 in the spinal cord.

### Neurophysiological interpretation

Elicited by stimulation of the peripheral nerves in the upper limb, an electrophysiological signal is transmitted along the sensory fibers, travels up dorsal column through the cervical segment of the spinal cord, and finally reaches the brainstem and cerebral hemispheres. It has been confirmed that the SEP components generated in the brainstem and in the cerebral cortex are mediated entirely by the dorsal columns (posterior columns) of the spinal cord, the fasciculus cuneatus for upper limb SEPs and the fasciculus gracilis for lower limb SEPs^3^. Therefore, various lesion sites along the dorsal column might lead to the absence of mediation effect of certain portions of the dorsal column on the SEP components, which consequently result in distinct distribution patterns of the SEP components in the time-frequency domain. In this study, pathological changes at different locations along the dorsal column were observed to produce morphological changes of SEP waveform and different SEP TFC distribution patterns, which verified this implication.

Results of this study infer that the somatotopic regions disturbed by the injury might lead to different recruitment of cortex, producing differences of SEP TFCs. A previous study^25^ on spinal cord resting state fMRI suggested that the propriospinal connections is not uniformly distributed across cervical spinal cord, while C5 showed a higher degree of connectivity in comparison to C4 and C6. This could be the reason that classifier II differentiated the C5 group from C4 and C6 injury groups based on middle-energy TFCs.

A previous study proposed that certain small components of the SEP could be generated from the far-field potentials within the cervical spinal cord^26^. It was reported that lesions involving the dorsal column could lead to prolongation or polyphase of selected small components in SEPs^9^. Thus, changes of these potentials could possibly reflect the location of pathological changes in the spinal cord. Previous findings are likely to correspond to the latency and/or power of the middle-energy TFCs in this study (Fig. 5(b) and Fig. 6) because most of these TFCs were found to be present with a short latency prior to the main component of SEP. This finding suggests that detailed analysis of SEPs could be used to explore useful information with respect to the pathological site of SCI.

A series of negative wavelets were identified near the major negative potentials (N1 in Fig. 4) with an approximate latency range from 18 to 30 ms. Some of these wavelets with relatively higher energy contributed to the formation of N1, while whereas others with lower energy were probably associated with selected subcomponents in previous research^9, 10^ according to their latency and relative energy. These wavelets were generated independently at different cortical regions^10, 27^. In this study, eight middle-energy TFCs (Fig. 5(b)) and eight low-energy TFCs (Fig. 5(c)) were found to fall in this temporal range (also near N1), and both of them show location-dependent characteristics. However, based on the limited number of TFCs found in this temporal range, one cannot yet draw the conclusion that the time-frequency distribution patterns of these TFCs (or the corresponding subcomponents in previous studies mentioned above) are also related to the pathological location of SCI. This phenomenon must be further demonstrated using a larger number of animal subjects in future work.

A set of low-frequency middle-energy TFCs appeared after 30 ms (Fig. 5(b)) and constituted the W-shape middle-latency response in the SEP waveform, although the shape became obscure after the appearance of lesions in the spinal cord caused by compression. These TFCs were presumed to be the subsequent waves evoked by the preceding strong nerve response^19^. Additionally, a number of small fluctuations were superimposed on (or affiliated with) these features, which corresponded to the low-energy TFCs in this temporal range (Fig. 5(c)) whose origins remained unclear. Difference of the distribution patterns of these TFCs could be found among the three injury groups. Based on this finding, the decision boundaries of Classifier II and Classifier III were estimated, which inferred that these TFCs might also contain information on the location of pathological changes after SCI. However, further investigation is still needed for a better understanding of the origins of these TFCs and the underlying pathological mechanism of the changes of their distribution pattern after chronic SCI.

### Selection of time points for data collection

The neuropathological process after SCI in rats is shorter than in humans^28^. Previous experiments showed that pathological changes in the spinal cord occurred in the first week after implantation^29^ and continued to progress over 2 weeks. When the compression lasted for more than 2 weeks, pathological changes began to spread to adjacent levels and became a multi-level pathological situation. Thus, the observation time point of two weeks was selected in this study, which can be viewed as a relatively stable period of pathological changes limited to the single level.

### Classification method

Without a relative energy threshold (2% in this study), i.e., using all of the small components apart from the main components in SEPs, it was found that a ‘one-stop’ classifier cannot be constructed to distinguish among the three injury groups (C4, C5, and C6). A multi-class SVM was attempted, but the classification performance was poor. The rationale for classifying the decomposed TFCs into three categories was based on a previous study^19^, which also decomposed SEPs (in the normal condition) into components in the time-frequency domain and applied a relative energy threshold of 2%. In the current study, we found that above this threshold, the TFCs in C5 group showed a distribution pattern distinct from those in the other two groups, and below this threshold, the TFC patterns of the other two groups, i.e., C4 and C6, had different distribution areas in the time-frequency domain. Based on this preliminary observation, the current three-step classifier was developed.

Theoretically, a multi-stage classifier with many stages is similar to a decision-tree learning method. By increasing the number of stage/levels, the classification accuracy might increase in a single training and testing round, but at the same time, the over-fitting problem is more likely to occur, which might reduce its generalization ability as reflected by a higher sensitivity to a new input and a lower average cross-validation accuracy. Therefore, we only used a 3-stage method in the current study.

The selection of a relative energy threshold was based on a previous study^19^ in which the 2% threshold was used for the first time. Findings in this rat study showed that the relative energies of the high- and middle-energy TFCs accounted for major proportion of the whole SEP signal. All the remaining low-energy TFCs had relative energies lower than 1%. If using 2% as the threshold, the middle- and low-energy TFCs could be clearly differentiated. The same phenomenon can also be observed in another study focusing on human SEP data^30^. In this previous human study, high- and middle-energy TFCs had large relative energy, while the remaining low-energy TFCs also have relative energy below 1%. According to this published report, the threshold of 2% could be applied to human data with a slight change that would not significantly affect the performance of the proposed classification method. Therefore, the 2% threshold was used in the current study to differentiate between middle- and low-energy TFCs.

It should be noted that the concept of classification in this study could be translated to humans. When implementing the classification method in humans, the same classifier would still be usable with a minor adjustment to the threshold for differentiating the middle- and low- energy TFCs. This means that the 2% threshold used in rat data might require a slight alteration for applicability in human data.

Lastly, the effect of SVM parameters (C and gamma) on the result of classification is discussed as follow. The classification result was not very sensitive to the varying of the parameter C. When log_2_(C) changed around the optimal value, the decline rate of classification accuracy was within 10%. But changes in gamma had effect on the performance of SVM. When log_2_(gamma) changed from the optimal value, the maximal decline rate of classification accuracy reached 30%. Therefore, the selection of gamma should be within a small range around the optimized value: [log_2_(gamma_0_)−1, log_2_(gamma_0_) +1], where gamma_0_ is the optimized value of gamma determined by the process of cross-validation.

### Relationship between pathological changes and classification performance

Table 1 shows quantitative measurements of pathological changes before and after spinal cord compression. Significant differences between the normal and injured conditions were found, which are probably correlated with the distributions of SEP TFCs of the normal and injury groups (Fig. 5(a)). This difference further contributes to the performance (an accuracy of 78.7%) of Classifier I.

Because the animal model used in this study was a mild compression model, the performance of this method should lie within the range corresponding to mild compressive SCI. Under this condition, the location-related information contained in SEPs was more easily affected by irrelevant components or noise than in the condition of more severe compression, which was probably the cause of the errors (misclassification) made by the developed classifier.

### Clinical implications

In clinical practice, damage to the spinal cord is evaluated by radiographic evaluation and neurologic examination. Radiographic tests including CT, X-rays, and MRI allow the morphological abnormality of the spinal cord and spinal canal to be inspected and the anatomy of cervical spine to be shown but do not offer detailed information on the pathological changes and deficits in neurological function of the spinal cord. Previous studies show that the correspondence between anatomical abnormalities and the existence of neurological impairment remains contentious^31–33^. The neurological examination can offer information on the status of neurologic function, but it cannot serve as an accurate diagnosis of the responsible level^34^. Cortical SEP signals have been widely used in intraoperative monitoring because they directly access the functional integrity of the central nervous system^35^. The findings in this study broaden the use of clinical SEP detection. In conventional clinical applications of SEP (such as clinical diagnosis in patients with neurologic diseases and intraoperative monitoring during surgeries that place portions of the somatosensory pathways at risk), only the main peaks (such as N20 in upper limb SEPs and P37 in lower limb SEPs) were investigated, and their temporal domain parameters (latency and amplitude) were used as an assessment of the functional integrity of the sensory pathway. The other components were considered as noise or unknown information. However, the current study is the first to explore the useful meaning in these small time-frequency components. The distribution pattern of these time-frequency components was found to reflect the location of pathological changes following SCI, which could be used as a new clinical test of SEP signal.

The proposed technique could be used in monitoring the rehabilitation process after SCI which is often very challenging due to the low sensitivity of standard outcome metrics. In some clinical cases, main component of the SEP signal may be absent from observation. The pathological condition of SCI therefore could hardly be evaluated. The proposed method could identify the smaller components apart from the main component. These small components could provide useful information regarding the pathological changes during the process of rehabilitation. Therefore, this technique could probably improve the current situation by increasing the sensitivity of SCI evaluation.

### Limitations and future scope

In future studies, the concept of SEP-based level diagnosis will be implemented in clinical human data. Previous studies^19, 30, 36, 37^ have shown that the components in rat SEPs are similar to those in humans, which infers that the distribution pattern of the TFCs in human SEPs might also be used as an indicator of the SCI pathological site. A classification method applicable to human data will be designed and tested.

Moreover, in addition to the three locations involved in this study (C4, C5, and C6), abnormalities might also occur at C2, C3, and/or C7 in human patients with a certain (although smaller) probability^33^. An analysis of the SEP patterns corresponding to those locations should be conducted such that the function of identifying these locations can be added to the current method.

Clinically, multilevel cervical myelopathy is quite common. A precise diagnosis of the pathological locations caused by multilevel compression is difficult because no consistent one-to-one match between anatomic abnormalities and neurologic impairment is available. If the specific time-frequency patterns of SEPs can be applied, it would be beneficial for the surgeon to understand the single-level and multi-level neurological deficits and select the most appropriate treatment.

## Conclusion

In this work, SEP signals were decomposed into detailed components using a high-resolution time-frequency analysis technique. Joint probability density estimation was applied to obtain the stable distributions of these components as defined by their time-frequency features. These time-frequency features were found to be able to reflect the pathological sites of SCI. By applying the machine learning method, the current study established an SEP-based classification method for identifying the location of pathological changes caused by cervical SCI. In this study, meaningful information contained in various SEP components was investigated and used to propose a new application of SEPs to identify the location of pathological changes in the cervical spinal cord. The developed classification method offers a non-invasive method of ascertaining the pathological level for preoperative diagnosis and postoperative assessment in cervical myelopathy or chronic SCI.
